# The IL‐1/IL‐1R axis induces greater fibroblast‐derived chemokine release in human papillomavirus‐negative compared to positive oropharyngeal cancer

**DOI:** 10.1002/ijc.31852

**Published:** 2018-11-26

**Authors:** Sarmad Al‐Sahaf, Keith D. Hunter, Robert Bolt, Penelope D. Ottewell, Craig Murdoch

**Affiliations:** ^1^ School of Clinical Dentistry, Claremont Crescent University of Sheffield United Kingdom; ^2^ Department of Oncology & Metabolism, Medical School, Beech Hill Road University of Sheffield United Kingdom

**Keywords:** head and neck cancer, human papillomavirus, fibroblast, tumour microenvironment, cytokine, leukocyte

## Abstract

Human papillomavirus (HPV) is now recognised as a major aetiological agent in the pathogenesis of oropharyngeal carcinoma (OPC). HPV‐positive tumours are associated with better outcomes compared to HPV‐negative tumours, possibly due to differences in their aetiology and/or the tumour microenvironment. Increased numbers of tumour‐associated leukocytes have been observed in many cancers including OPC, with variable influence on prognosis depending on the leukocyte subpopulation investigated. Whether HPV status influences leukocyte recruitment to OPC remains unknown. This *in‐vitro* study examined differences in the chemoattractant capacity of HPV‐positive and HPV‐negative OPC cell lines. Gene and protein expression analysis demonstrated that whilst both monocultures of HPV‐positive and HPV‐negative cell lines, along with normal tonsillar fibroblasts (NTF), expressed low chemokine levels, NTF cultured with conditioned medium from HPV‐negative OPC cells expressed significantly higher levels of all chemokines tested compared to NTF incubated with the medium from HPV‐positive OPC cell lines. HPV‐negative OPC lines expressed IL‐1β mRNA whereas HPV‐positive cells did not, and NTF constitutively expressed IL‐1R1. Pre‐treatment with the IL‐R antagonist, anakinra or siRNA to IL‐1R1 significantly reduced chemokine secretion from NTF stimulated with conditioned medium from HPV‐negative tumour cells or recombinant IL‐1β (*p* < 0.05). These data suggest that secretion of chemokines is driven by the interaction between HPV‐negative OPC cells and stromal fibroblasts through an IL‐1/IL‐1R‐mediated mechanism that is less prominent within the HPV‐positive tumour microenvironment. These observations may explain differences in leukocyte sub‐populations recruited to HPV‐positive *versus* negative OPC and indicate that HPV status is a key determinant in controlling the inflammatory tumour microenvironment.

## Abstract 2

**What's new?**

Rates of oropharyngeal cancer (OPC) are increasing, mainly due to human papillomavirus (HPV) infection. There is limited understanding, however, about the affects of HPV status in OPC on tumor‐stromal interactions, which influence tumor development and progression. In this study, HPV‐negative OPC cells were found to drive a robust chemokine secretion profile in stromal tonsillar fibroblasts, whereas HPV‐positive OPC cells exhibited relatively low chemokine expression. Increased chemokine expression in stromal tonsillar fibroblasts was mediated by the IL‐1/IL‐1R axis. The findings suggest that HPV status plays a role in the recruitment of leukocyte sub‐populations in the OPC tumor microenvironment, influencing inflammatory activity.

**Abbreviations**
ANOVA: analysis of variance
ELISA: enzyme‐linked immunosorbent assay
FBS: fetal bovine serum
HLA: human leukocyte antigen
HNC: head and neck cancer
HPV: human papillomavirus
IL: interleukin
MAPK: mitogen‐activated protein kinase
MTT: (3‐(4,5‐Dimethylthiazol‐2‐yl)‐2,5‐Diphenyltetrazolium Bromide)
NTF: normal tonsillar fibroblast
OPC: oropharyngeal carcinoma
PBS: phosphate‐buffered saline
PCR: polymerase chain reaction
SCC: squamous cell carcinoma

## Introduction

Head and neck cancers (HNC) are the sixth most common cancer globally and ninth most common malignancy in males in the United States.^1^ These cancers are distributed throughout the head and neck region, although much recent attention has been paid to oropharyngeal carcinoma (OPC) due a disproportionate increase in incidence over the past 10 years.^2^, ^3^, ^4^ The aetiology of OPC appears to be multi‐factorial and includes not only tobacco use and alcohol consumption, but also human papillomavirus (HPV) infection,^4^, ^5^ which now accounts for up to 65% of all OPC cases in the United States^6^ and 50% in the United Kingdom.^4^

Several studies have consistently demonstrated that individuals with HPV‐positive OPC display better overall survival compared to their HPV‐negative, stage‐matched counterparts.^7^, ^8^, ^9^ HPV‐positive tumours also display an improved response to surgery, chemotherapy and radiotherapy when compared to HPV‐negative tumours.^10^, ^11^ The reasons for these differences remain largely unknown, although it is generally accepted that the favourable prognosis of HPV‐positive OPC is related to the progressive genetic aberrations acquired in HPV‐negative cancers, including key cell cycle regulators such as TP53 and RB1, whereas HPV‐positive tumours arise from the effects of viral oncoproteins E6 and E7, which allow tumour cells to retain a wild‐type TP53 and RB1 status.^12^ Given the complex cellular interactions that drive tumourigenesis; it is likely that disruption of other pathways are also important in promoting the development of HPV‐positive and ‐negative OPC.

The tumour microenvironment is instrumental in the progression of many cancers, particularly with respect to inflammation.^13^, ^14^ In HPV‐negative HNC, the presence of high numbers of tumour‐associated macrophages and neutrophils has been associated with poor prognosis.^15^, ^16^, ^17^ In addition, HPV‐negative OPC has been shown to recruit immunosuppressive, regulatory T‐cells that may suppress any cytotoxic anti‐cancer responses.^18^ Recent evidence suggests that HPV‐positive tumours preferentially attract CD8 T‐cells, which, if present in high numbers, are predictive of an improved outcome.^19^ Together these findings suggest that the variation in leukocyte populations recruited to HPV‐positive *versus* HPV‐negative tumours may have important connotations for outcome and prognosis.^20^

Tumour‐infiltrating leukocyte recruitment is mainly driven by a large family of chemotactic cytokines (chemokines) that are often up‐regulated in cancer.^21^, ^22^ These molecules direct the movement of specific leukocyte subpopulations from the circulation into the tumour milieu, with the nature of the infiltrating leukocyte population, and therefore inflammatory burden, being dependent on the composition of chemokines released. Although OPC cell lines are able to secrete a number of chemokines, there is increasing evidence that it is the crosstalk between tumour cells and stromal fibroblasts that is more important for cancer progression.^14^ We have recently demonstrated that HPV‐negative cancer cells drive the secretion of hepatocyte growth factor from oral fibroblasts which, in turn, influences tumour cell invasion; a mechanism that is reduced in response to HPV‐positive cells.^23^ It is therefore plausible that the chemotactic cues driven by the tumour microenvironment in HPV‐positive OPC is different from those in HPV‐negative OPC and this may account for differences observed in tumour‐associated leukocyte sub‐populations. However, to date there is little data on how tumour cell HPV status may impact on chemokine expression in OPC. Therefore, our study evaluated the expression of chemokines within an *in vitro* model of HVP‐positive and HPV‐negative tumour/stromal cell interactions to determine if HPV status has an influence on the molecular drivers of leukocyte recruitment in OPC.

## Materials and Methods

### Cell culture

OPC cells UPCI‐SCC89, UPCI‐SCC72 (HPV‐negative) and UPCI‐SCC90, UD‐SCC2 (HPV‐positive) were received through material transfer agreement with Professor Susanne Gollin, University of Pittsburgh. Hypopharyngeal carcinoma cell lines used included FaDu (HPV‐negative) and UPCI‐SCC152 (HPV‐positive). Cell lines were authenticated by DNA short tandem repeat analysis by the Genomics Core Facility at the University of Sheffield and HPV status confirmed by the HPV Cytology Screening Unit (Sheffield Teaching Hospital NHS Foundation Trust, UK). Normal tonsillar fibroblasts (NTF) were isolated from patients during routine tonsillectomies undertaken at the Royal Hallamshire Hospital, Sheffield Teaching Hospitals NHS Foundation Trust with written, informed consent^24^ (ethical approval 09/H1308/66). SCC89, SCC72, SCC90 cells and NTF were cultured in Dulbecco's Modified Eagle's Medium and SCC2, FaDu, SCC152 were grown in RPMI, all supplemented with 10% v/v FCS, 2 mM l‐glutamine, 100 IU penicillin and 100 μg/mL streptomycin in a humidified incubator with 5% CO_2_, at 37 °C. All materials were purchased from Sigma‐Aldrich, UK, unless otherwise stated. Most studies were performed on NTF06 (female, age 29); however, additional studies to confirm consistency across NTF from different donors were also performed (NTF01 (female, age 38), NTF319 (male, age 52) and NTF322 (female age 21)).

### Generation of tumour cell conditioned medium

Tumour cell lines were seeded at 1 × 10^7^ cells in 75cm^2^ flasks for 24 h in serum‐containing medium, washed three times with phosphate‐buffered saline (PBS) then incubated with 6 mL serum‐free medium for 24 h. Conditioned medium was collected, centrifuged at 400g for 5 min to remove cell debris and stored at −80 °C. NTF were grown to 80% confluence in 75cm^2^ flasks, washed three times with PBS and then incubated with either cancer cell line conditioned media or serum‐free medium for 24 h. Cancer cell+NTF or NTF alone conditioned medium was then retrieved, centrifuged at 400g for 5 min and stored at −80 °C for later analysis. For IL‐1R blocking experiments, 6 × 10^5^ NTF were incubated with 10 μg/mL anakinra or placebo control (Amgen, Thousand Oaks, CA) for 2 h before stimulation with conditioned medium from SCC89 OPC cells or medium containing 5 ng/mL recombinant IL‐1β as a positive control for 4 h. Levels of anakinra or placebo were maintained throughout the experiment. A cytotoxicity assay using the tetrazolium dye MTT was performed on anakinra treated NTF as previously described.^25^

### siRNA treatment of NTF

siRNA for IL‐1R1 was performed according to the manufacturer's instructions (FlexiTube, Qiagen). Briefly, 3 × 10^5^ NTF seeded in 6‐well plates were incubated with transfection complex containing 300 nM siRNA (IL‐1R1, oligonucleotide SI00017598) and Hi‐PerFect transfection reagent (both Qiagen) in serum‐free medium. The transfection complexes were added drop‐wise to cells and the tissue culture plates swirled to ensure equal distribution of the transfection complex. Mock‐transfected control cells received transfection reagent alone. Cells were incubated for 24 h at 37 °C in 5% CO_2_ with serum‐containing medium before washing twice with PBS and addition of conditioned medium from SCC89 cells. All medium was collected after 4 h and analysed for chemokine content by ELISA.

### Chemokine protein arrays and ELISA

Human chemokine arrays (C1 series, RayBiotech, USA) were used to screen the medium from NTF cultured with conditioned medium derived from HPV‐positive or ‐negative OPC cell lines, according to the manufacturer's instructions. The relative expression levels of each chemokine were determined by densitometry using Quantity One software (Biorad, USA). Commercially available ELISA kits for CXCL8, CCL2 (BD Biosciences) and CCL5 (R&D systems) were used to quantify protein levels according to the manufacturer's instructions.

### RNA isolation, reverse transcription and quantitative polymerase chain reaction (RT‐qPCR)

Total RNA was isolated (BioLine, UK) and 500 ng reverse transcribed (High‐Capacity kit, Life Technologies, UK). qPCR was performed using Taqman gene expression assays as follows: 0.5 μL cDNA was amplified using 5 μL TaqMan master‐mix, 3.5 μL nuclease‐free water and 0.5 μL TaqMan gene probes for CXCL1 (Hs00236937), CXCL5 (Hs01099660), CXCL8 (Hs00174103), CCL2 (Hs00234140), CCL5 (Hs00982282), CCL7 (Hs00171147), IL‐1α (Hs00174092) and IL‐1β (Hs00174097), 0.5 μL β‐2‐Microglobulin (Hs00984230) was used as a reference control (Applied Biosystems). Reactions were performed using thermal cycles of 50 °C (2 min) and 95 °C (10 min) then 40 cycles of 15 s at 95 °C followed by 1 min at 60 °C. The threshold cycle (Ct) was normalised against the reference gene and then fold changes in expression relative to the untreated NTF group calculated by using the formula 2^‐ΔΔCt^. For expression of IL‐1α and IL‐1β by cancer cells, gene expression was normalised to the abundance of the control gene transcript, β‐2‐microglobulin.

### Immunofluorescence staining of NTF

NTF were seeded on glass coverslips at 1 x10^4^ cells in 24‐well plates and incubated overnight before being fixed and permeabilised using 2% paraformaldehyde and 0.1% Triton‐X‐100 for 15 min. After blocking with bovine serum albumin for 30 min, cells were incubated with 5 μg/mL of either anti‐IL‐1R1 (R&D Systems, clone 73,229), HLA class I (Sigma‐Aldrich, clone W6/32) or IgG control antibody (Abcam) at 4 °C overnight. After washing with PBS, cells were incubated with biotin‐conjugated goat anti‐mouse antibody (Abcam) for 30 min then with phycoerythrin‐conjugated streptavidin for 20 min at room temperature. after washing and mounting with ProLong™ Diamond anti‐fade containing 4′,6‐Diamidine‐2′‐phenylindole dihydrochloride (DAPI), cells were imaged using a Zeiss880 AiryScan confocal microscope.

### Statistics

All data are expressed as mean ± SD of at least three independent experiments unless otherwise stated. Statistical analysis was performed using GraphPad Prism‐v7.0 (GraphPad Software). Group‐wise comparisons were carried out using one‐way independent ANOVA with Tukey's multiple comparisons test and differences were considered significant when *p* < 0.05.

## Results

### HPV‐positive and HPV‐negative‐stimulated NTF display differential chemokine expression

**Figure 1:**
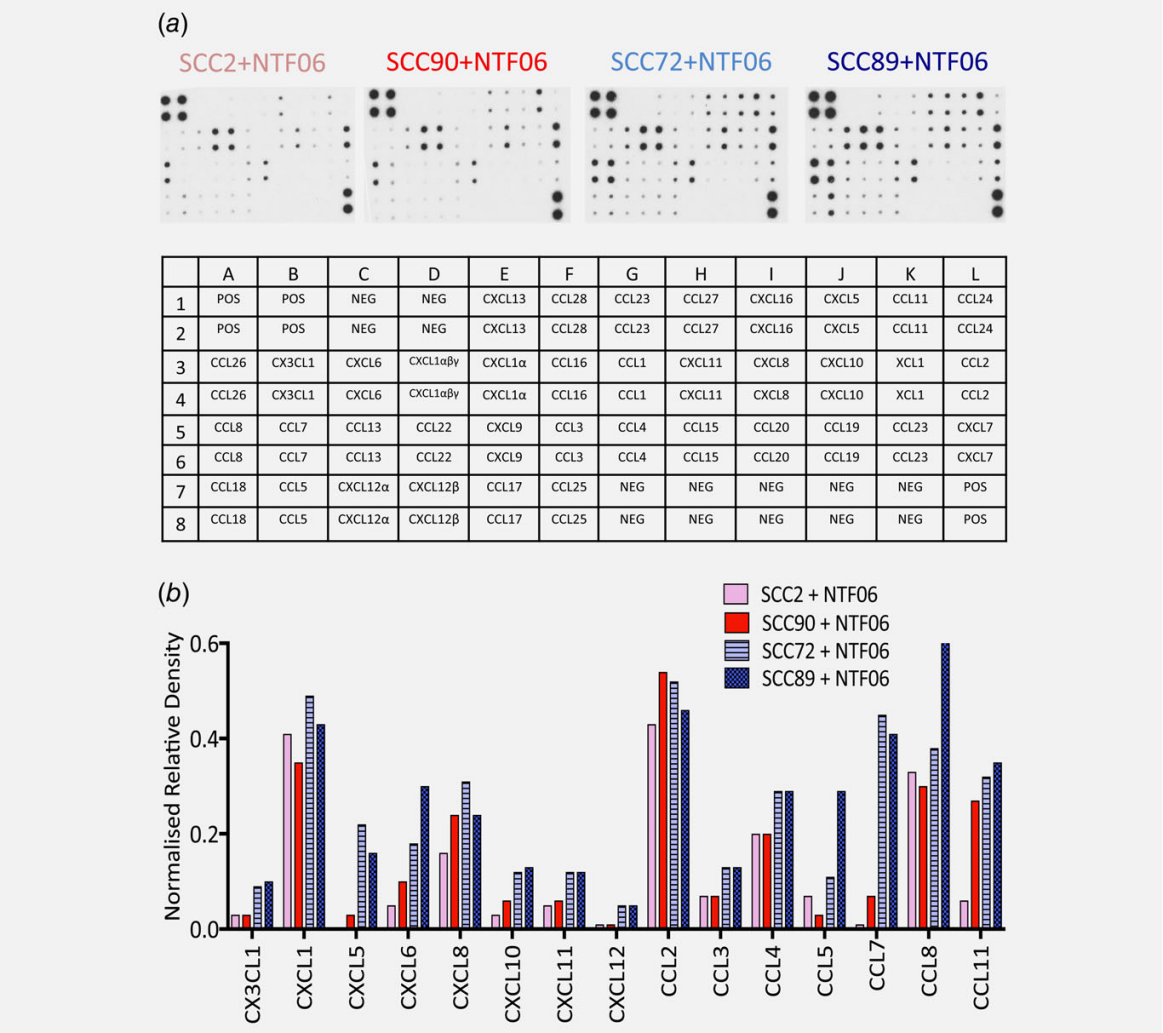
Normal tonsillar fibroblasts (NTF), cultured with conditioned medium from HPV‐negative OPC, secrete increased levels of chemokines compared to HPV‐positive OPC (*a*) Chemokine arrays were probed with conditioned medium from NTF06 incubated with SCC2 and SCC90 (HPV‐positive) or SCC72 and SCC89 (HPV‐negative) conditioned medium. (*b*) Bar chart shows densitometry analysis of arrays where immunoblots were scanned and the density of the spots normalised to the intensity of the positive control spots to allow for comparisons. Data shown are from one representative blot of two experimental repeats. [Color figure can be viewed at wileyonlinelibrary.com]

**Figure 2:**
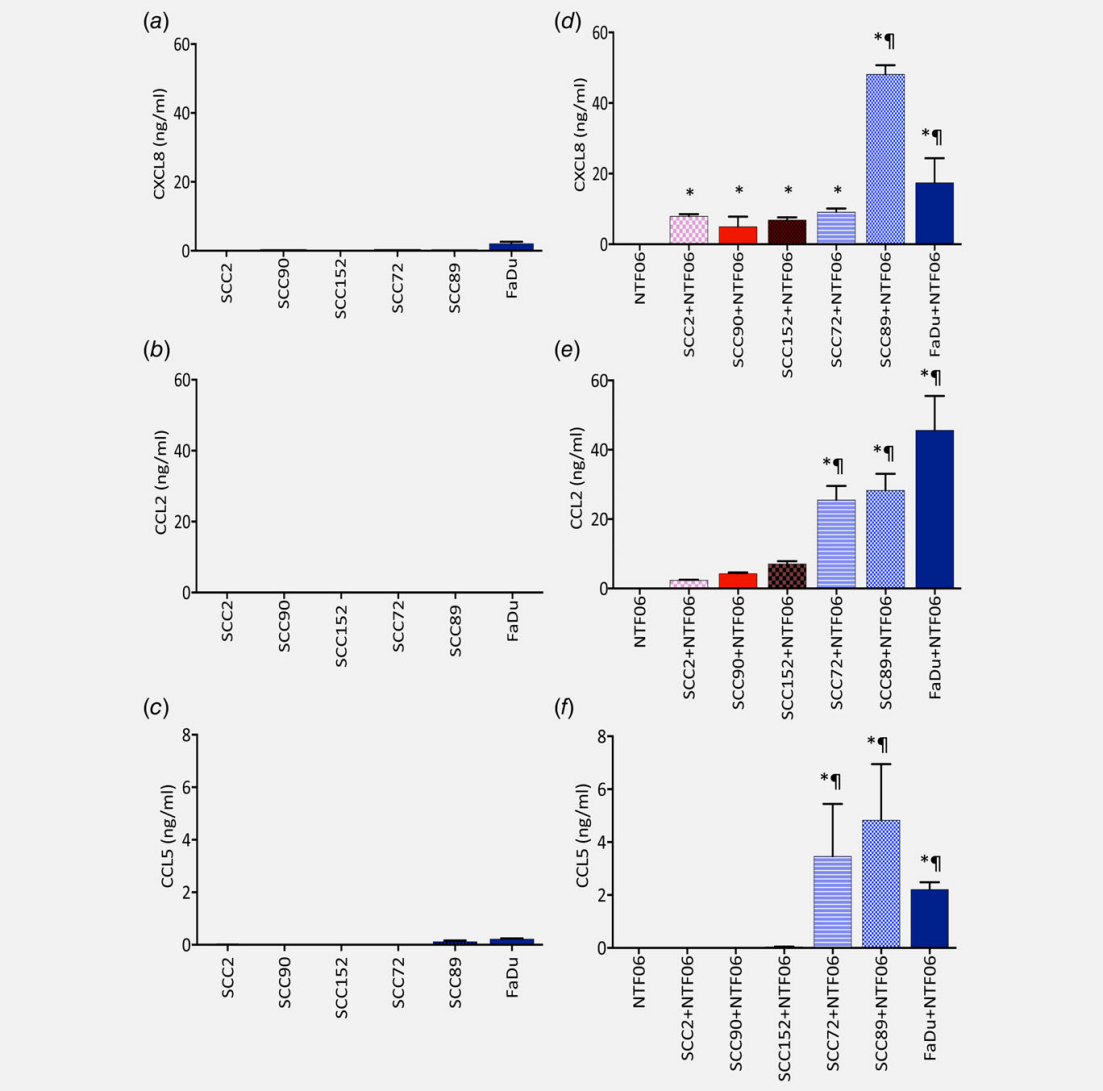
HPV‐negative conditioned medium stimulates markedly increased chemokine release from NTF than HPV‐positive conditioned medium. Chemokine release was quantified by ELISA in the conditioned medium of HPV‐positive (SCC2, SCC90, SCC152) or HPV‐negative (SCC72, SCC89, FaDu) tumour cells cultured alone for (*a*) CXCL8 (*b*) CCL2 (*c*) CCL5. NTF06 were cultured with the conditioned medium produced from either HPV‐positive (SCC2, SCC90, SCC152) or HPV‐negative (SCC72, SCC89, FaDu) tumour cells for 24 h and compared to NTF cultured alone for (*d*) CXCL8, (*e*) CCL2 and (*f*) CCL5. Data are mean ± SD from at least 3 independent experiments performed in triplicate and statistical analysis was achieved using a one‐way independent ANOVA with Tukey's post‐hoc multiple comparison test. *at least *p* < 0.05 compared to NTF alone; ¶ *p* < 0.01 compared to SCC2, SCC90 and SCC152. [Color figure can be viewed at wileyonlinelibrary.com]

Chemokine protein array profiling showed that the conditioned medium from the HPV‐negative OPC cell lines SCC72 and SCC89 induced a robust and marked increase in overall chemokine secretion by NTF06 compared to SCC2 and SCC90 HPV‐positive cells (Fig. 1). Semi‐quantitative densitometric analysis showed abundant secretion of a broad spectrum of CXC, CX3CL and CCL chemokines for HPV‐negative‐stimulated NTF06, with particularly large increases for neutrophil‐specific (CXCL1, −5, −6, −8), monocyte‐specific (CCL7, −8) and T‐lymphocyte (CCL3, −4, −5) chemokines. Although the protein array data gives an idea as to the overall chemokine burden induced by HPV‐positive/negative tumour cell/stromal interactions, it is neither quantitative nor reveals the cell origin of chemokine production. Therefore, we used ELISA for a subset of leukocyte chemokines ‐ CXCL8 (neutrophils), CCL2 (monocytes) and CCL5 (T‐cells) to determine whether cancer cells or NTF were the main chemokine producers. Both HPV‐negative and HPV‐positive tumour cells and NTF06 alone express extremely low levels of all chemokines tested (Fig. 2 *a*,*b*,*c*). Upon stimulation with conditioned medium from HPV‐positive cancer cells (SCC2, SCC90, SCC152) the amount of chemokine in the conditioned medium was increased compared to NTF06 alone, but was still relatively low. In contrast, incubation of NTF06 with conditioned medium derived from HPV‐negative cancer cells (SCC72, SCC89 and FaDu) significantly increased the amount of CXCL8 (SCC89 *p* < 0.001 and FaDu *p* < 0.01), CCL2 and CCL5 (at least *p* < 0.05 for all HPV‐negative cells) in the conditioned medium (Fig. 2 *d*,*e*,*f*). For SCC89‐treated NTF06, the increase in CXCL8 was time‐dependent, with the levels of this chemokine still increasing after 24 h (Supporting Information Fig. S1). To show that this stimulatory effect was not due to use of NTF06 alone, NTF isolated from 3 other donors (NTF01, NTF319 and NTF322) were stimulated with the conditioned medium from HPV‐positive SCC2 or HPV‐negative SCC72 OPC cells. These data show that, although some donor‐to‐donor variation was observed, HPV‐negative SCC72 conditioned medium consistently induced significantly (*p* < 0.01) greater chemokine secretion from all the NTF tested than for HPV‐positive SCC2 conditioned medium (Supporting Information Fig. S2). The increased chemokine secretion by these NTF in response to SCC72 conditioned medium was much reduced compared to the levels observed for SCC89 conditioned medium (Figs. 1 and 6), showing that differences in NTF chemokine secretion is HPV‐negative cell line dependent.

### HPV‐negative, but not HPV‐positive, conditioned medium induces chemokine gene expression by NTF

**Figure 3:**
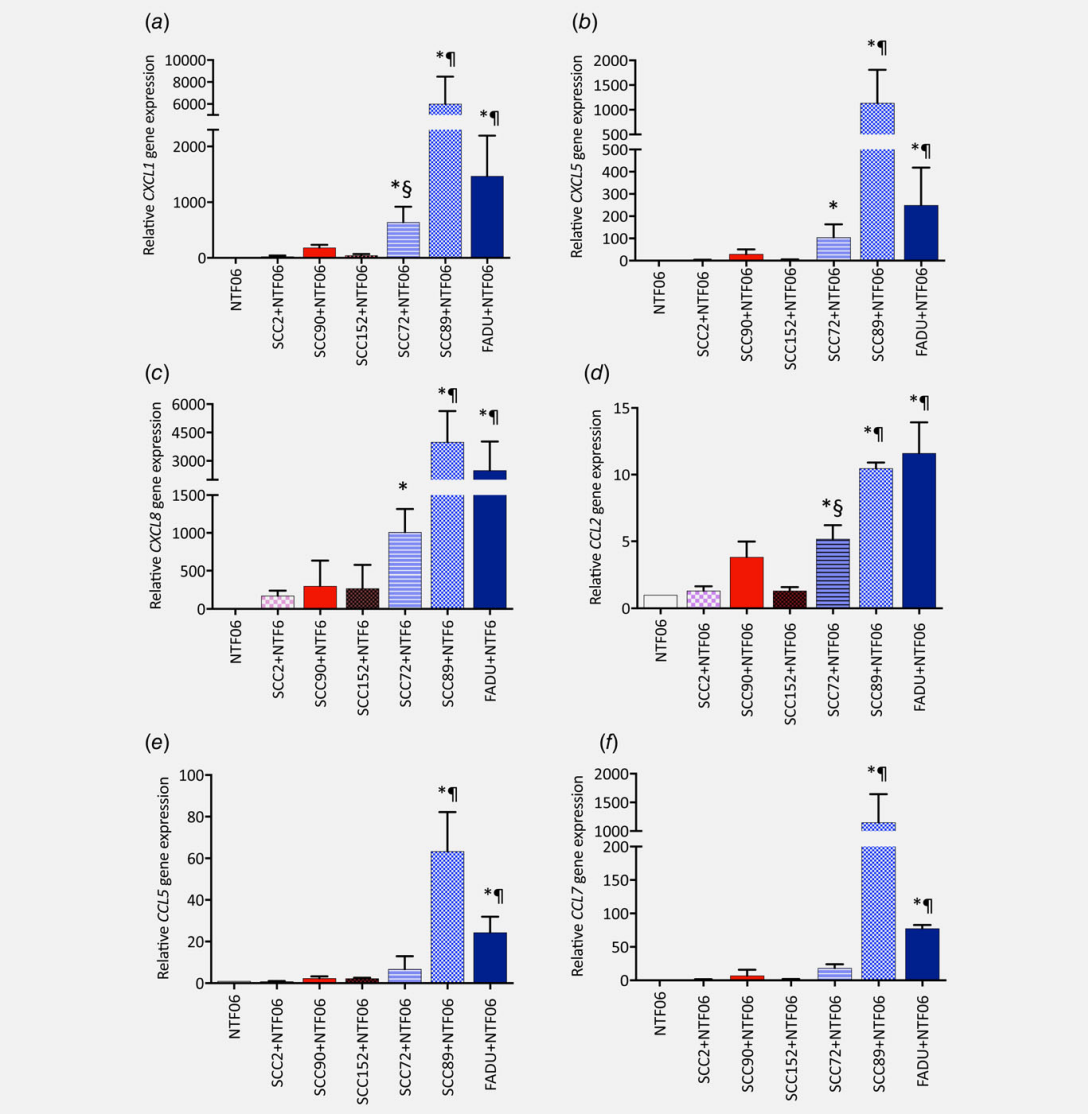
HPV‐negative conditioned medium stimulates increased chemokine gene expression from NTF06 than HPV‐positive conditioned medium. NTF were cultured with the conditioned medium from either HPV‐positive cells (SCC2, SCC90, SCC152) or HPV‐negative (SCC72, SCC89, FaDu) for 24 h or NTF06 cultured alone and gene expression analysed by qPCR for (*a*) CXCL1, (*b*) CXCL5, (*c*) CXCL8, (*d*) CCL2, (*e*) CCL5 and (*f*) CCL7. Data are presented as mean fold‐change in gene expression ± SD compared to NTF06 alone from 3 independent experiments performed in triplicate and statistical analysis was achieved using a one‐way independent ANOVA with Tukey's post‐hoc multiple comparison test. *at least *p* < 0.05 compared to NTF alone; ¶ at least *p* < 0.05 compared to SCC2, SCC90 and SCC152; § *p* < 0.05 SCC72 compared to SSC2 and SCC152. [Color figure can be viewed at wileyonlinelibrary.com]

Quantitative PCR was next performed to determine whether induction of chemokine expression was extended to the mRNA level. Expression of genes encoding the neutrophil‐specific chemoattractants, CXCL1, CXCL5 and CXCL8 were all significantly (*p* < 0.05) increased when NTF06 were cultured with conditioned medium derived from HPV‐negative compared to NTF06 treated with HPV‐positive‐derived conditioned medium or NTF06 cultured with medium alone (Fig. 3 *a*–*c*). Similar observations were found for genes encoding the monocyte‐specific chemokines CCL2 and CCL7 (Fig. 3 *d*,*f*). In addition, the gene encoding the T‐cell chemokine CCL5 was also significantly increased by HPV‐negative conditioned medium but only for SCC89 and FaDu cells (Fig. 3 *e*). In all cases tested, the level of gene expression was directly related to cell type in terms of the ability to induce chemokine production by NTF06 with SCC89 < FaDu<SCC72. Taken together, these data suggest that HPV‐negative cancer cells secrete factor(s) that induce chemokine gene, and in turn protein, expression by NTF and that the factor(s) are largely absent from HPV‐positive conditioned medium.

### IL‐1β is expressed by HPV‐negative but not HPV‐positive cancer cells

**Figure 4:**
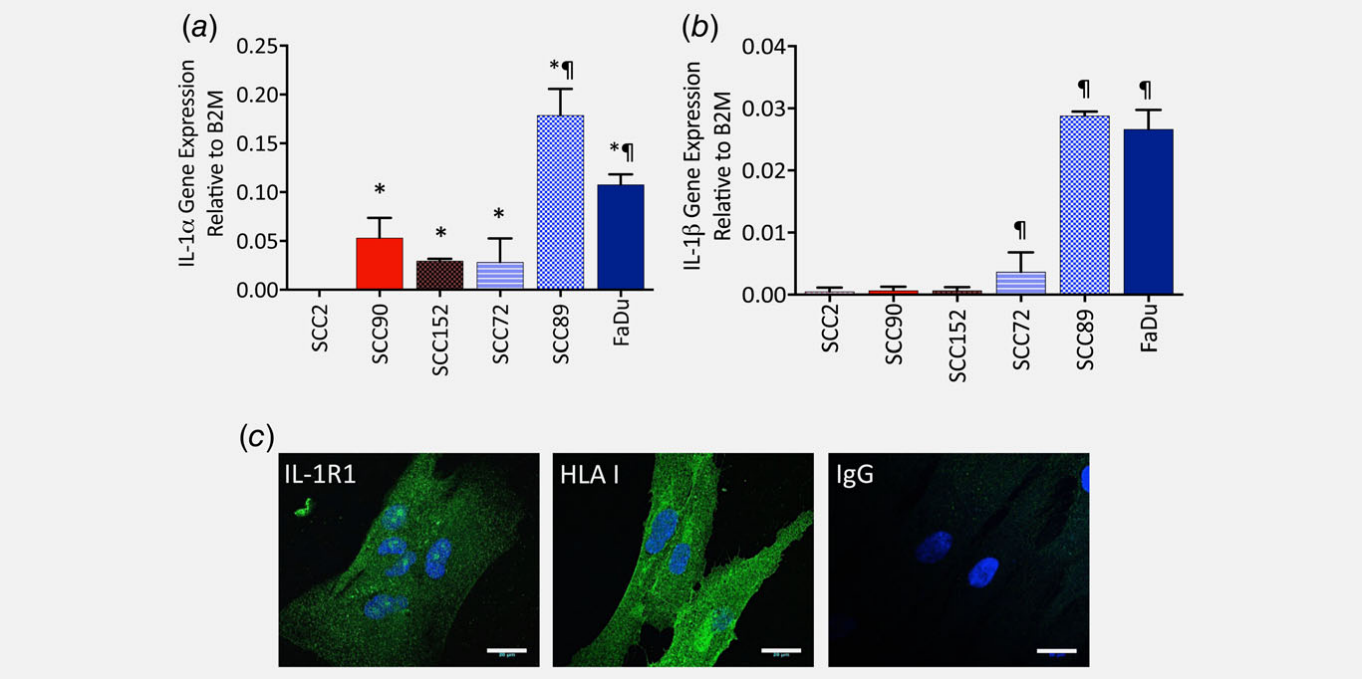
HPV‐negative tumour cells express abundant IL‐1α and IL‐1 β gene transcripts. Gene expression of (*a*) IL‐1α and (*b*) IL‐1β by HPV‐positive (SCC2, SCC90, SCC152) or HPV‐negative (SCC72, SCC89, FaDu) tumour cells was quantified by qPCR. Expression of IL‐1α and IL‐1β was normalised to the internal reference control gene, β‐2‐microglobulin to provide measures of relative expression for each cell line. Data presented from 3 independent experiments performed in triplicate and statistical analysis was achieved using a one‐way independent ANOVA with Tukey's post‐hoc multiple comparison test. *at least *p* < 0.05 compared to SCC2; ¶ at least *p* < 0.05 compared to SCC2, SCC90 and SCC152. (*b*) Expression of IL‐1R1 on NTF06 by immunofluorescence staining. Cells were fixed, permeabilised and stained with a monoclonal antibody for IL‐1R1, HLA class I (positive control) or IgG (negative control) and visualised by confocal microscopy. NTF displayed cell surface and intracellular IL‐1R1 expression. Data show images from one NTF but are representative of 3 independent experiments. [Color figure can be viewed at wileyonlinelibrary.com]

Chemokine gene expression is predominantly induced by pro‐inflammatory cytokines, usually by activation of the MAPK and NFκB signalling pathways.^26^ This prompted us to examine the expression of pro‐inflammatory cytokines by HPV‐positive and HPV‐negative OPC cells. Neither HPV‐positive nor HPV‐negative cells expressed tumour necrosis factor‐α (data not shown). In contrast, IL‐1α gene expression was detected in both HPV‐positive and HPV‐negative cells, although gene expression was significantly elevated in the HPV‐negative lines SCC89 and FaDu (4‐ and three‐fold, respectively) compared to all HPV‐positive cell lines (*p* < 0.01, Fig. 4 *a*). Differential expression of IL‐1β was even more evident; with HPV‐positive cells expressing negligible levels, whilst HPV‐negative cells all expressed abundant IL‐1β transcripts, with highly significant increases observed for SCC89 and FaDu (*p* < 0.01, Fig. 4*b*). We further conducted a preliminary study using biopsy tissue sections from HPV‐positive and HPV‐negative OPC to examine IL‐1β expression *in vivo* by immunohistochemical analysis. We found that IL‐1β was minimally expressed in normal tonsillar epithelium. In contrast, the presence of IL‐1β was generally observed in most HPV‐negative OPC tumour cells, whereas expression was not observed in most HPV‐negative OPC tumour cells. However, staining was variable amongst sections and the presence of IL‐1β was observed in tumour sections from certain HPV‐positive biopsies (Supporting Information Fig. S3).

Both IL‐1α and IL‐1β potentiate their pro‐inflammatory actions by binding to IL‐1R expressed by target cells. Immunofluorescence staining of NTF06 cells using a specific anti‐human IL‐1R1 antibody followed by confocal microscopy analysis showed punctate expression of IL‐1R1 in NTF, providing evidence of both cell surface and intracellular receptor expression (Fig. 4 *c*). A similar staining pattern was observed for NTF isolated from different donors (Supporting Information Fig. S4).

### IL‐1/IL‐1R axis mediates NTF chemokine expression by HPV‐negative cancer cells

**Figure 5:**
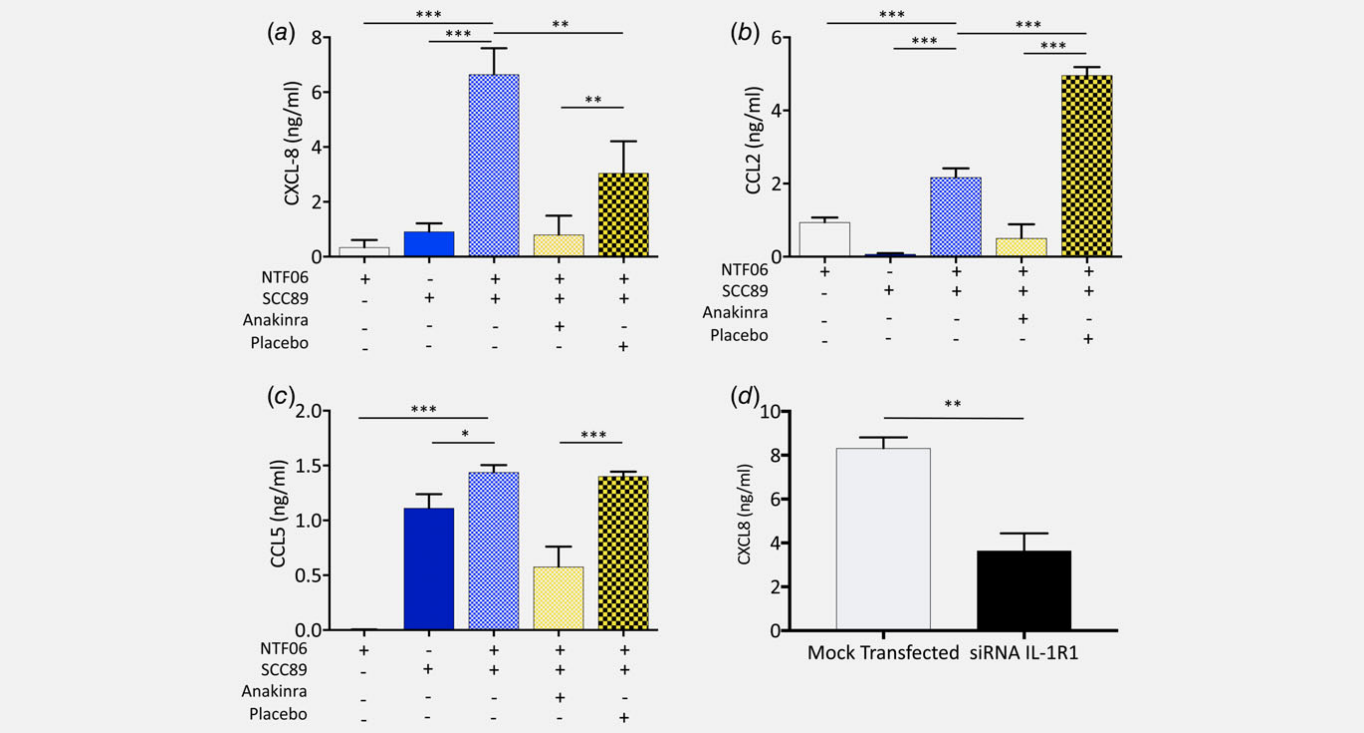
NTF chemokine release by HPV‐negative conditioned medium is driven by an IL‐1/IL‐1R1 axis. (*a*) NTF06 cells were incubated with anakinra or placebo then stimulated with the conditioned medium from SCC89 tumour cells for 4 h and levels of chemokines (*b*) CXCL8, (*c*) CCL2 and (*d*) CCL5 quantified in the medium by ELISA and compared to levels of these chemokines from NTF06 incubated with the conditioned medium from SCC89 cells without treatment, or the conditioned medium from SCC89 or NTF cells cultured alone. In each case chemokine secretion was significantly decreased by pre‐incubation with the IL‐1R antagonist anakinra compared to placebo control. (*d*) NTF06 were treated with siRNA to ablate IL‐1R1 expression and then stimulated with conditioned medium from SCC89 cells. siRNA‐treated Cells exhibited a significant decrease in CXCL8 secretion compared to mock‐transfected NTF06 cells. Data are mean ± SD from at least 3 independent experiments performed in triplicate. Statistical analysis for *a*, *b* and *c* was achieved using a one‐way independent ANOVA with Tukey's post‐hoc multiple comparison test. In panel D, statistical analysis was a Mann–Whitney U test for 4 independent experiments. *p < 0.05, **p < 0.01, ***p < 0.001. [Color figure can be viewed at wileyonlinelibrary.com]

**Figure 6:**
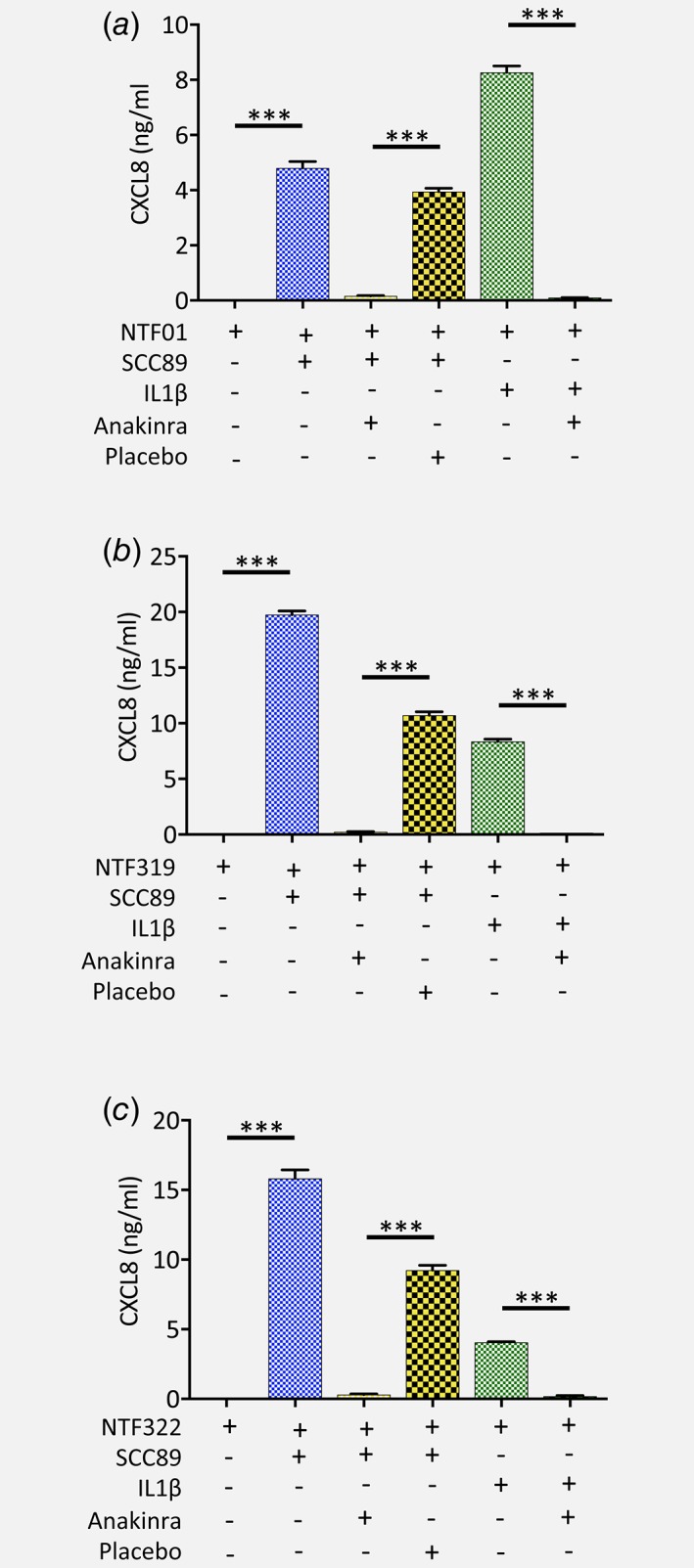
IL‐1/IL‐1R1 axis increases chemokine secretion by NTF isolated from different donors. (*a*) NTF01, (*b*) NTF319 and (*c*) NTF322 were pre‐treated with anakinra or placebo then stimulated with the conditioned medium from SCC89 tumour cells or 5 ng/mL IL‐1β and levels of CXCL8 in the conditioned medium quantified by ELISA and compared to levels of this chemokine from NTF incubated with the conditioned medium from SCC89 cells without treatment, or the conditioned medium from SCC89 or NTF cells cultured alone. In each case CXCL8 secretion was significantly decreased when pre‐incubated with the IL‐1R antagonist anakinra compared to placebo control. Data are mean ± SD from 3 independent experiments performed in triplicate. Statistical analysis was achieved using a one‐way independent ANOVA with Tukey's post‐hoc multiple comparison test. ***p < 0.001. [Color figure can be viewed at wileyonlinelibrary.com]

To determine whether the IL‐1 expressed by HPV‐negative cells was responsible for inducing chemokine release *via* IL‐1R, NTF06 were incubated with conditioned medium derived from SCC89 cells in the presence or absence of the highly specific IL‐1R1 antagonist anakinra, and levels of CXCL8, CCL2 and CCL5 secreted quantified by ELISA. Medium from SCC89 cells was used in these experiments because these cells were identified as expressing most abundant IL‐1 as well as generating a robust chemokine release profile by NTF. An MTT cytotoxicity assay was initially used to confirm that neither anakinra nor the placebo control were toxic to NTF in concentrations up to 100 μg/mL (Supporting Information Fig. S5). As with previous data, both NTF and SCC89 when incubated alone expressed minimal CXCL8 and CCL2, whereas NTF06 incubated in SCC89 conditioned medium significantly induced the secretion of both chemokines (Fig. 5 *b*,*c*). Pre‐incubation of NTF06 with anakinra significantly (*p* < 0.01) abrogated secretion of CXCL8 and CCL2, reducing levels to that of unstimulated NTF06. Interestingly, pre‐incubation with the placebo significantly (*p* < 0.01) reduced levels of CXCL8 whilst increasing levels of CCL2 (*p* < 0.01) although in both instances the levels of these chemokines were still significantly higher than that detected after anakinra treatment (*p* < 0.01, Fig. 5 *a*,*b*). The pattern of CCL5 expression differed to that of CXCL8 and CCL2. Overall, levels of CCL5 were much lower than those observed for CXCL8 or CCL2. CCL5 was detected in SCC89 medium alone, although secretion significantly increased when the media was incubated with NTF06 (*p* < 0.05). Treatment with anakinra significantly (*p* < 0.01) reduced CCL5 levels, although not back to baseline, whilst treatment with placebo had no effect (Fig. 5 *c*). To confirm specificity, IL‐1R1 gene expression by NTF06 was reduced using gene specific siRNA oligonucleotide probes (expression reduced by 88 ± 3% when analysed by qPCR). When incubated with SCC89 conditioned medium, secretion of CXCL8 was significantly reduced from 8.0 ± 0.9 ng/mL in mock‐transfected NTF06 to 3.6 ± 0.9 ng/mL (*p* < 0.01) in siRNA treated cells (Fig. 5 *d*). To determine that this phenomenon was not due to one individual NTF batch, we repeated the anakinra blocking experiment with NTF derived from 3 separate donors and tested for CXCL8 release in response to SCC89 conditioned medium. Treatment of all batches of NTF with SCC89 conditioned medium led to a significant increase in CXCL8 secretion (*p* < 0.001). In addition, pre‐incubation with anakinra resulted in a total abrogation of CXCL8 chemokine secretion (*p* < 0.001) in response to conditioned medium derived from SCC89 cancer cells for all NTF tested that was not evident in the placebo control (Fig. 6). Moreover, treatment of these NTF with recombinant IL‐1β also induced a prominent CXCL8 response that was likewise completely inhibited by the IL‐1R antagonist (Fig. 6).

## Discussion

Despite the often late diagnosis of HPV‐positive OPC and lymph node involvement, prognosis is significantly better than that of its HPV‐negative counterpart.^27^ It has been proposed that this improved prognosis is related, in part, to differences in the immune response of these tumours,^28^ which, by extension, may be related to levels of tumour‐infiltrating leukocytes. However, data relating to the nature of leukocyte‐recruiting chemokines released from HPV‐positive and HPV‐negative OPC is lacking. The tumour microenvironment involves crosstalk between cancer and stromal cells^13^, ^29^ and this interaction appears to be crucial for many aspects of cancer progression.^23^, ^30^ We therefore used a 2D *in vitro* model to examine HPV‐positive *versus* HPV‐negative cancer‐stromal interactions, and how such interactions may influence micro‐environmental chemokine release.

Chemokine protein array data demonstrated that the conditioned medium from HPV‐negative OPC when incubated with NTF contained increased amounts of chemokines encompassing most chemokine family members. Several previous reports have shown that OPC cells have the ability to express chemokines,^31^, ^32^, ^33^ indeed, our quantitative analysis showed that CXCL8, CCL2 and CCL5 were all expressed by both HPV‐negative, HPV‐positive and NTF but at very low levels. However, secretion of these chemokines along with others were substantially increased when all NTF examined were stimulated with the conditioned medium from HPV‐negative, but not HPV‐positive cells. Moreover, gene expression analysis showed that HPV‐negative conditioned medium was able to activate the transcription of several chemokine genes by NTF, whereas HPV‐positive conditioned medium did not. The chemokine response by NTF was cell line dependent with some HPV‐negative cells lines stimulating more chemokine expression than others, suggesting some level of heterogeneity between these cells that may be dependent upon their genetic background. These data not only show differential activation of chemokine genes in response to conditioned media depending on tumour line HPV‐status, but also point to a pro‐inflammatory cytokine as the molecular trigger.

The most studied and potent of inflammatory mediators belong to the TNF and IL‐1 families. We found transcripts for IL‐1α and IL‐1β preferentially in HPV‐negative cells. Indeed, IL‐1β was expressed exclusively in HPV‐negative OPC. Constitutive expression of IL‐1β has been detected previously in keratinocytes^34^ where levels increase upon immune stimulation or malignant transformation.^35^, ^36^ Moreover, Wu *et al*. showed that silencing IL‐1β with lentivirus‐delivered shRNA significantly inhibited oral squamous cell carcinoma cell growth both *in vivo* and *in vitro*, heavily implicating IL‐1β as a key regulator of the tumour microenvironment during HPV‐negative oral cancer development.^36^ Curiously, a complete lack of IL‐1β expression has been observed in HPV‐positive cervical carcinoma cells lines, implying HPV as a central driving force in IL‐1β ablation. Niebler *et al*., showed that infection of primary keratinocytes with HPV16 E6, but not E7, abrogated IL‐1β processing and secretion in a proteasome‐dependent manner that was mediated by ubiquitin ligase E6‐AP and p53.^35^ The authors further suggest that continual IL‐1β ablation leads to complete loss of IL‐1β gene altogether as HPV‐mediated malignancy develops.^35^ It is tempting to speculate that a similar mechanism also exists in HPV‐positive OPC cells and this may explain the difference in IL‐1β expression between *in vitro* cultured HPV‐positive and HPV‐negative OPC cells observed in our study. Indeed, in preliminary immunohistochemical studies on a small number of human HPV‐negative and HPV‐positive OPC biopsies, we found, in general, that HPV‐negative tumour cells appeared to express more abundant levels of IL‐1β than HPV‐positive tumour cells. However, we also observed IL‐1β expression by some HPV‐positive tumour cells, suggesting that loss of IL‐1β *in vivo* is not absolute. It is plausible that expression levels of IL‐1β may differ significantly by HPV status *in vivo* and therefore a larger cohort study examining IL‐1β levels in HPV‐negative/positive OPC is warranted.

Although our study is the first to identify IL‐1R1 expression on tonsillar fibroblasts, this receptor has been detected previously on gingival fibroblasts.^37^ We used anakinra, a highly specific IL‐1R antagonist that has been used clinically to treat auto‐inflammatory diseases,^38^, ^39^ to block cancer cell derived IL‐1 binding to the NTF IL‐1R, to show that CXCL8, CCL2 and CCL5 release is driven in NTF by the IL‐1/IL‐1R axis, presumably *via* IL‐1β secreted by HPV‐negative OPC. In support of these findings, siRNA ablation of NTF IL‐1R1 also significantly reduced chemokine secretion in response to HPV‐negative SCC89 conditioned medium. Inhibition of chemokine secretion using siRNA to IL‐1R was not as effective as inhibition with anakinra. This may be due to more efficient blockade of IL‐1R by anakinra compared to siRNA treatment where IL‐1R was reduced by 88%, and so signalling may occur through the IL‐1R still expressed by si‐RNA treated cells. In addition, we used siRNA specific for IL‐1R1 and so IL‐1β may exert its actions on other IL‐R family members expressed by NTF. Our data provides evidence that OPC HPV status may radically influence tumour biology by altering the inflammatory microenvironment. Indeed, increased levels of IL‐1β have been found in the cell constituents of HPV‐negative compared to HPV‐positive OPC, supporting the observations in our study.^32^

One of the major roles of chemokines in tumour biology is to attract leukocytes to tumour tissue. Although no study has reported data specific to HPV‐positive/negative OPC, abundant tumour‐associated macrophages and neutrophils have been found in HPV‐negative tumours in animal models, and in human HNC where they have been linked to poor prognosis.^15^, ^16^, ^40^, ^41^ This may be due to the actions of tumour‐derived IL‐1β on stromal fibroblasts, as evidenced in our study. Moreover, once recruited, tumour‐associated leukocytes, in particular macrophages, may secrete additional IL‐1β further potentiating a feedback loop of chemokine release, leukocyte recruitment and inflammation within the tumour microenvironment that drives tumour progression.^42^ Although further studies are warranted, it is interesting to speculate that IL‐1/IL‐1R inhibitors may prevent accumulation of tumour‐associated myeloid cells, improving outcome. Although there are inherent problems with IL‐1R blockade, inhibition of IL‐1β has shown promising results in animal models of several cancers.^43^, ^44^

Whilst chemokines driving myeloid cell infiltration appear to promote tumour progression, there is evidence that recruitment of certain T‐cell populations is associated with improved prognosis. Indeed, in a prospective study, the presence of elevated levels of tumour‐infiltrating CD8+ T‐cells correlated with a good prognosis in HPV‐positive compared to HPV‐negative OPC.^19^, ^32^, ^45^, ^46^ Interestingly, we found levels of CCL5 as well as other T‐cell chemokines were elevated in HPV‐negative compared to HPV‐positive OPC stromal interactions. It is possible that CD8 cells may be recruited by chemokines or other factors not analysed in our study. CD8‐cell recruitment may be suppressed in HPV‐negative tumours even in the presence of elevated chemokines, possibly by the elevated presence of myeloid‐derived suppressor cells or regulatory T‐cells. Although representative OPC cell line panels were chosen for use in our study, the reported data cannot be readily generalised to clinical disease. 2D tumour/stromal interactions *in vitro* are not representative of tumours *in vivo* due to tumour hypoxia, nutrient exhaustion, and changes in vasculature supply. The data presented in our study will therefore need confirmation through *in vivo* and human cancer tissue studies.

In summary, we have found for the first time, that HPV‐negative OPC cells constitutively express IL‐1 that stimulates significant chemokine release from tonsillar fibroblasts *via* specific interaction with IL‐1R, whereas this molecular mechanism was less prominent in HPV‐positive OPC cells. IL‐1‐driven chemokine release may have a key role in the increased neutrophil and macrophage burden observed in HPV‐negative cancers, which is often associated with poor prognosis.

## Supporting information

Appendix S1: Supporting Information
