# Supplementary material for: The IL‐1/IL‐1R axis induces greater fibroblast‐derived chemokine release in human papillomavirus‐negative compared to positive oropharyngeal cancer
Source: Int J Cancer. 2018 Nov 26;144(2):334–44. doi: 10.1002/ijc.31852 (PMC6491969; doi:10.1002/ijc.31852)
Supplement: Supplementary file 1 — Appendix S1: Supporting Information [file IJC-144-334-s001.pdf]

Supporting Information

The IL-1/IL-1R axis influences fibroblast-derived chemokine release in human papillomavirus negative compared to positive oropharyngeal cancer

Sarmad Al-Sahaf, Keith D. Hunter, Robert Bolt, Penelope D. Ottewell, Craig Murdoch

[1] School of Clinical Dentistry, Claremont Crescent, University of Sheffield, S10 2TA, UK

[2] Department of Oncology & Metabolism, Medical School, Beech Hill Road, University of Sheffield, S10 2RX, UK

Supporting Figure S1

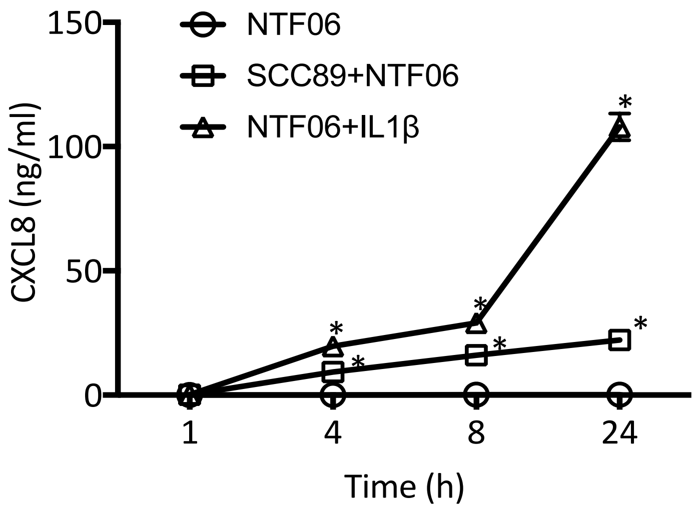

Figure S1. ***SCC89 HPV-negative conditioned medium stimulates increased CXCL8 secretion by NTF in a time-dependent manner***. NTF were either culture alone, stimulated with the conditioned medium from SCC89 cells or stimulated with 5 ng/ml IL-1β for up to 24 hours by then the NTF conditioned medium analysed for secretion of CXCL8 by ELISA. Data are presented as mean ± SD from at least 3 independent experiments performed in triplicate and statistical analysis achieved using a one-way independent ANOVA with Tukey's post-hoc multiple comparison test. *$p<0.05$ compared to NTF alone.

Supporting Figure S2

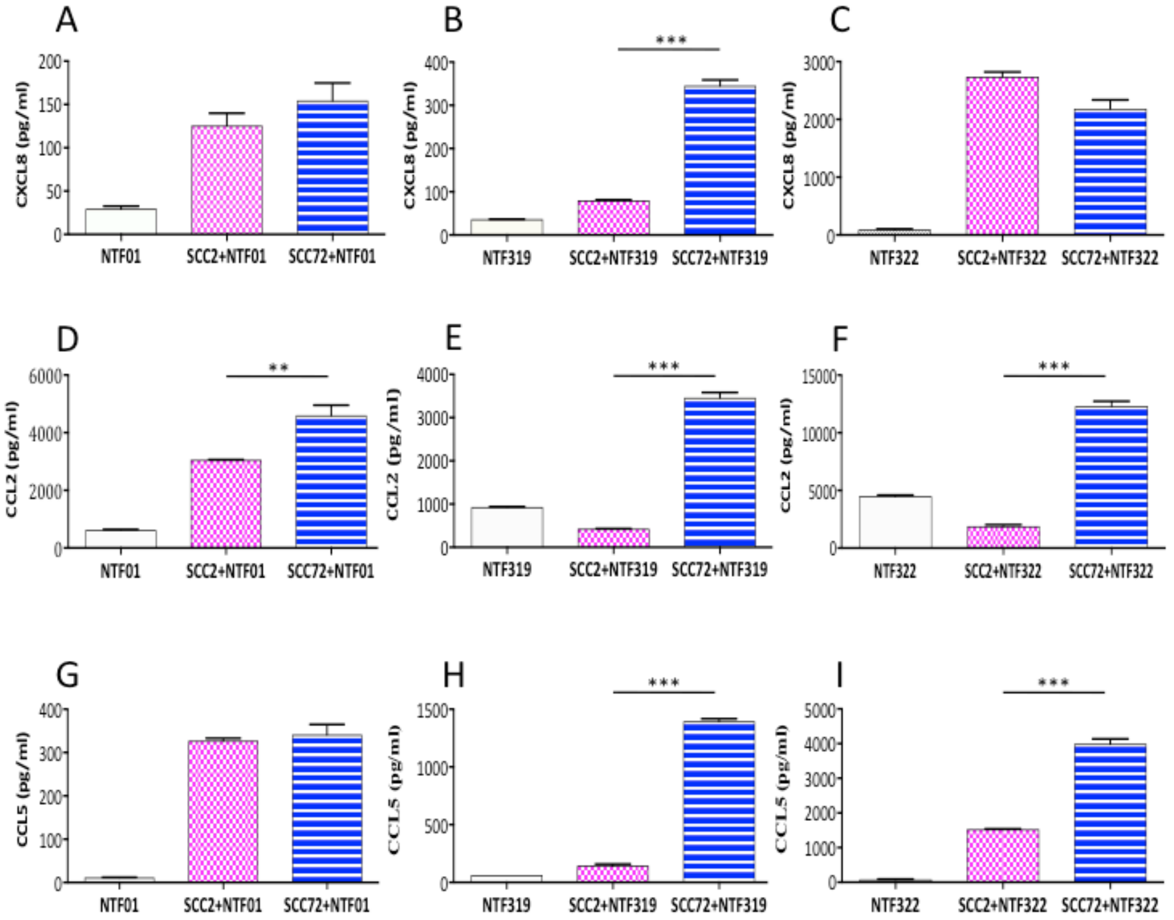

Figure S2. ***Conditioned medium form SCC72 HPV-negative OPC increases chemokine release by NTFs compared to SCC2 HPV-positive OPC cells.*** NTF isolated from different donors (NTF01, NTF319 and NTF322) were cultured with the conditioned medium form SCC72 HPV-negative or SCC2 HPV-positive OPC cells for 4 h and the levels of CXCL8 (A-C), CCL2 (D-F) and CCL5 (G-I) measured by ELISA. NTF cultured with medium alone was used as a control. In most cases the conditioned medium from HPV-negative SCC72 cells significantly increased chemokine release by each NTF compared to stimulation with HPV-positive SCC2 medium, although some NTF donor-to-donor variation was observed. Statistical analysis was a one-way independent ANOVA with Tukey's post-hoc multiple comparison test. $^{**}p<0.01$ $^{***}p<0.001$.

Supporting Figure S3

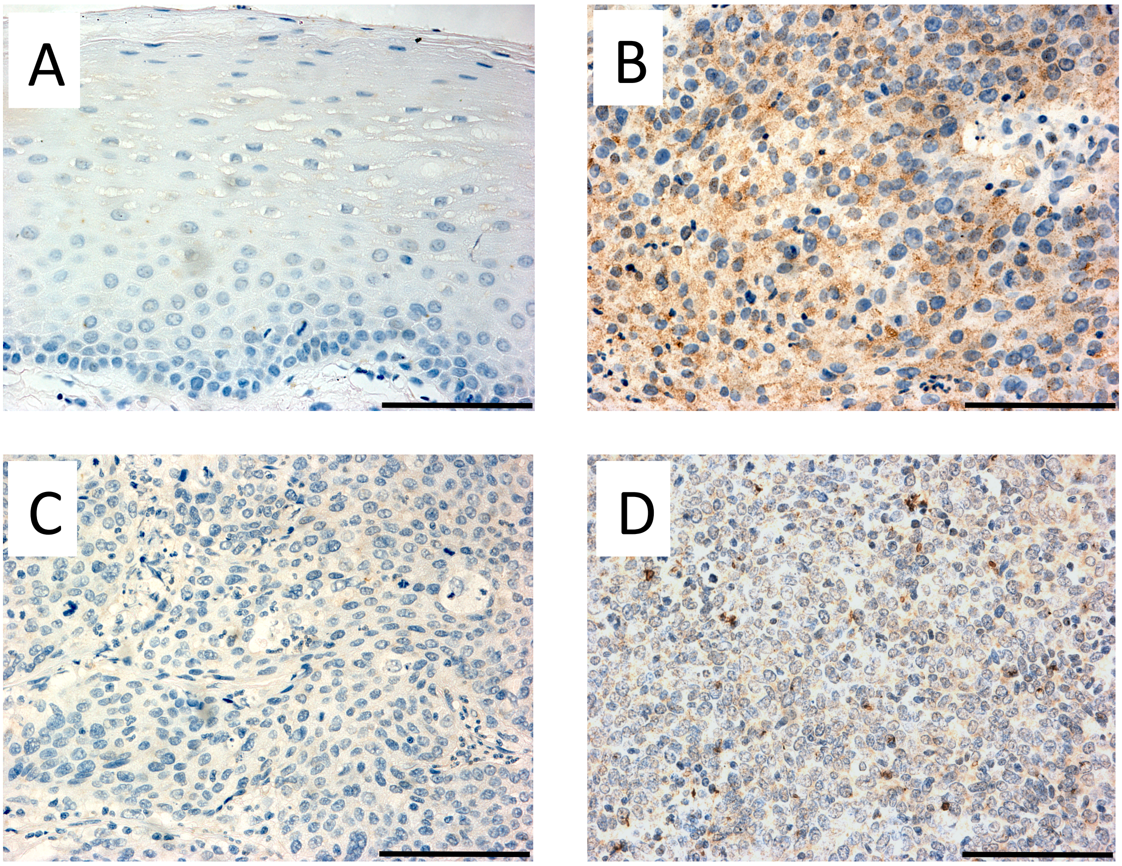

Figure S3. ***HPV-negative OPC express elevated levels of IL-1β compared to HPV-positive OPC.*** Paraffin-wax embedded tumour sections (5 µm) from confirmed HPV-negative OPC (n=5) and HPV-positive OPC (n=5) were stained by immunohistochemistry for IL-1β. Sections were de-waxed, dehydrated and high-temperature citrate buffer (pH6) antigen retrieval performed in a 2100 Antigen Retriever (Aptum Biologics Ltd). Following blocking, sections were incubated with anti-IL-1β antiserum (1:200 v/v; Ab2105, Abcam) in blocking buffer overnight at 4°C, washed with TBST and then a secondary antibody and avidin-biotin complex (Vector Labs) used in accordance with the manufacturer's instructions. 30-diaminobenzidine tetrahydrochloride (DAB) (Vector Labs) was used to visualise peroxidase activity and sections counterstained with haematoxylin, dehydrated and mounted in DPX. Images were taken using an Olympus BX51 microscope and Colour view IIIu camera with associated Cell^D software. IL-1β was minimally expressed in normal tonsillar epithelium (A). In general, IL-1β was observed in most HPV-negative OPC tumour cells (B) whereas expression was not observed in most HPV-negative OPC tumour cells (C). However, staining was variable amongst sections and the presence of IL-1β was observed in tumour sections from certain HPV-positive biopsies (D).

Supporting Figure S4

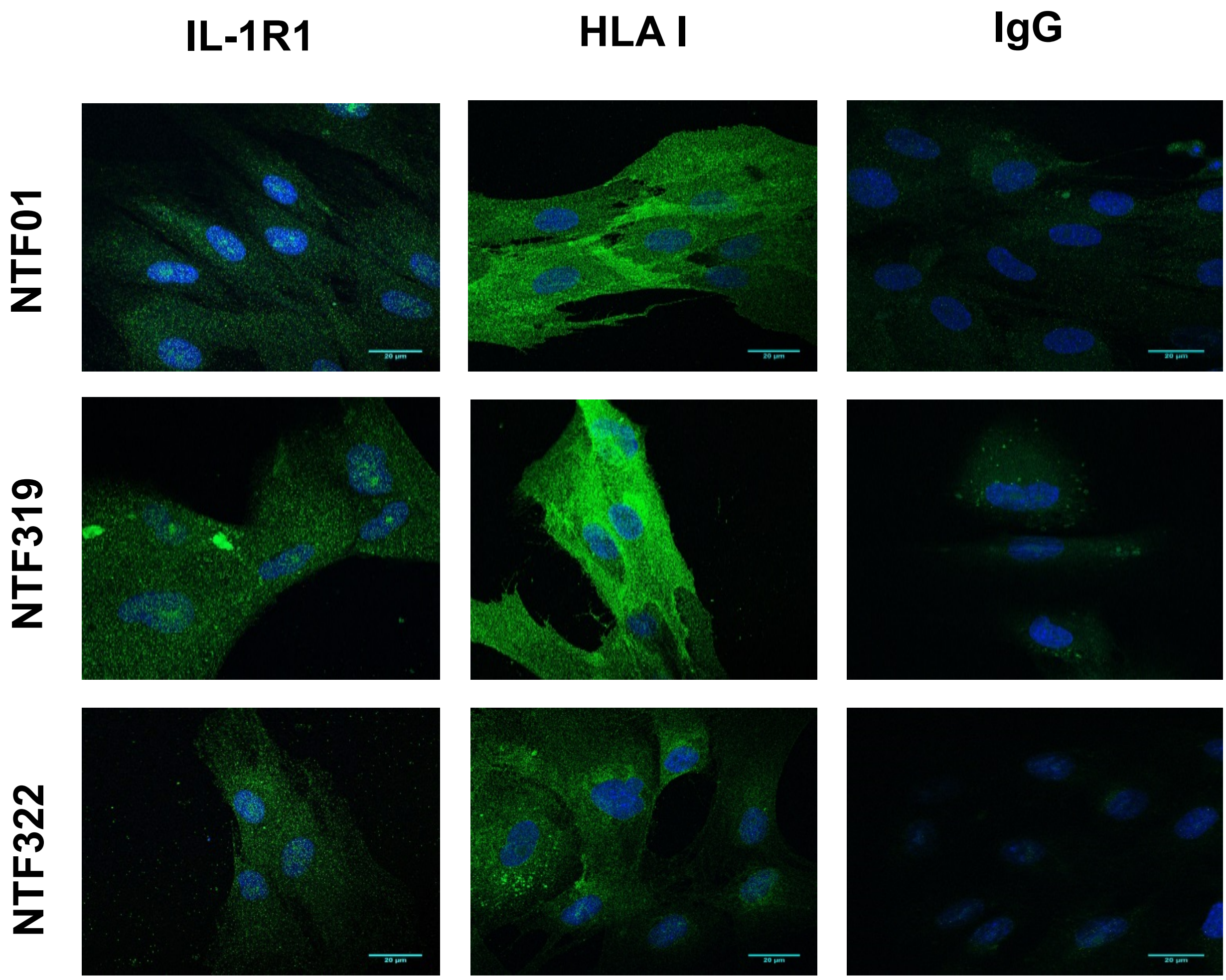

Figure S4. ***Expression of IL-1R1 by NTF01, NTF319 and NTF322 by immunofluorescence staining.*** NTF were fixed with 2% paraformaldehyde, permeabilised and stained with a monoclonal antibody for IL-1R1. Antibodies raised to HLA class I or IgG were used as positive and negative controls, respectively. Cell staining was visualised by confocal microscopy. NTF displayed cell surface and intracellular IL-1R1 expression.

Supporting Figure S5

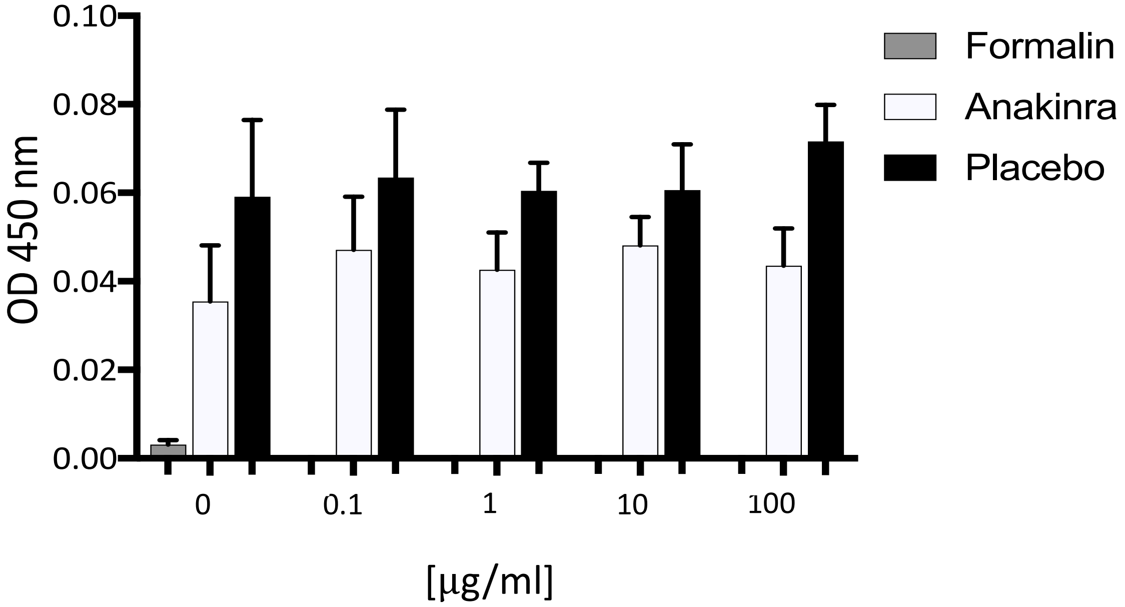

Figure S5. ***Anakina is not cytotoxic to NTF.*** NTF06 cells were treated with increasing concentrations of anakinra or placebo control and analysed for cytotoxicity using an MTT toxicity assay. Formalin-fixed cells were used as a positive control. There was no significant difference in metabolism between anakinra and placebo treated NTF cells. Data are mean ± SD for 3 independent experiments, each performed in triplicate.
